# Editorial: EEG rhythms: decoding the evolutionary enigma of alpha rhythms in vertebrates

**DOI:** 10.3389/fnhum.2025.1560294

**Published:** 2025-01-30

**Authors:** Takashi Shibata, Kaoru Takakusaki

**Affiliations:** ^1^Department of Neurosurgery, Toyama University Hospital, Toyama, Japan; ^2^Department of Neurosurgery, Toyama Nishi General Hospital, Toyama, Japan; ^3^The Research Center for Brain Function and Medical Engineering, Asahikawa Medical University, Asahikawa, Japan

**Keywords:** alpha rhythm, evolution, aging, emotion, cognitive load, psychosis, consumer behavior

## Introduction

The EEG alpha rhythm, oscillating at 8–13 Hz, has captured the attention of neuroscientists since its discovery in 1929 by Hans Berger (Vergani, 2024). This rhythm, often associated with wakeful rest and relaxation, plays a critical role in brain function. Despite decades of research, its evolutionary origins remain an enigma. We propose that the alpha rhythm played a pivotal role in the cognitive evolution of nocturnal mammals (Shibata et al.). By comparing mammalian brains with those of fish, reptiles, and birds, we have identified unique alpha rhythm characteristics in mammals. Future studies might offer further insights into its role in the evolution of advanced cognition, particularly in humans. Beyond its evolutionary implications, the alpha rhythm has been widely studied for its applications in health, clinical settings, and social contexts. This editorial highlights findings from five recent EEG studies that explore its diverse roles and implications in humans.

## Healthy individuals: aging, emotional experiences, and cognitive load

### Aging and triple-correlation EEG

Watanabe et al. explored age-related differences in alpha rhythms using triple-correlation EEG analysis. They discovered significant spatial (S values) and temporal (SD values) variability differences between young and older adults. Young individuals showed higher spatial variability (S values), reflecting a rich presence of normal cortical dipoles, especially in the frontal region. However, their temporal variability (SD values) was lower, indicating greater stability in alpha rhythm generation compared to older adults. Interestingly, young individuals' S values resembled those of dementia patients, but the addition of SD values differentiated “youthful dipoles” from “aging” and “pathological dipoles.” This innovative dipole concept provides a nuanced understanding of neurophysiological changes with age and offers a basis for distinguishing healthy aging from early signs of dementia.

### Emotional experiences and EEG microstates

Gupta et al. investigated the alpha rhythm's role in emotional processing using EEG microstate analysis and eLORETA. Participants viewed emotionally charged music videos categorized as “happy” or “sad.” During happy music, there was an increase in class D microstate occurrence and current source density (CSD) in the central parietal region, indicating enhanced attention. Conversely, sad music elevated class C microstate occurrence and functional connectivity in the precuneus, associated with increased mind-wandering. These findings underscore the alpha rhythm's involvement in emotional regulation and attention, revealing individual and gender-specific differences in brainwave responses to emotions.

### Cognitive load and infra-slow fluctuations of alpha power

Sazuka et al. demonstrated the alpha rhythm's importance in cognitive functions related to bodily states. Using a novel metric—infra-slow fluctuations of alpha power—they found that higher levels correlated with more efficient information processing and cognitive resource availability. Additionally, heart rate variability (HRV), a marker of parasympathetic nervous system activity, positively correlated with task accuracy. These results highlight the infra-slow fluctuations of alpha power as a marker of cognitive efficiency under load and its potential link with autonomic nervous system functioning.

### Clinical applications: predicting psychosis in at-risk mental state patients

Resting-state EEG studies have shown that schizophrenia patients exhibit reduced alpha power (especially α2) and increased δ and β1 power. Higuchi et al. extended this research to individuals at risk of developing psychosis (ARMS patients). Their findings revealed that those who later developed psychosis (ARMS-P) had significantly higher β1 power in the left middle frontal gyrus (MFG). Moreover, the β1/alpha power ratio positively correlated with physical anxiety scores, linking anxiety to psychosis risk. Given its accessibility and clinical relevance, β1 power is proposed as a biomarker for early psychosis prediction. Further research is needed to refine resting-state EEG patterns, but these findings highlight the potential of resting EEG power as a non-invasive, cost-effective diagnostic tool for early intervention.

### Social applications: consumer behavior and N400 responses

Gorin et al. explored the role of brainwaves in social contexts, with a particular focus on consumer behavior. Using EEG and MEG analyses, they identified neural responses resembling the N400, observed in reaction to mismatches between prices and products. In the MEG experiments, these responses were localized in the ventromedial prefrontal cortex (vmPFC) and the anterior cingulate cortex (ACC), areas involved in value-based decision-making. Unlike the traditional N400 response elicited by semantically incongruent words, these N400-like responses reflect distinct neural mechanisms underlying consumer judgment processing. Previous research has shown that trial-by-trial fluctuations in pre-stimulus alpha power can predict linguistic N400 responses (Lago et al., 2023). Building on this, further understanding the relationship between N400 neural markers and pre-stimulus alpha power could help businesses optimize pricing strategies to align with consumer expectations, thereby improving decision-making processes and customer satisfaction.

## Conclusion: evolutionary significance and multifaceted role of the alpha rhythm

The alpha rhythm, unique to mammals, might play a role in maintaining nocturnal wakefulness and is a critical physiological phenomenon for understanding the brain's functional and evolutionary complexity. This rhythm shows promise as a biomarker of brain activity, with broad implications—including aging, emotional processing, cognitive load, mental disorders, and social behavior. Fully understanding its evolutionary origins and practical applications will require interdisciplinary research that integrates insights from neuroscience, psychology, neuromarketing, and evolutionary biology. Such collaborative efforts have the potential to enhance our understanding of the brain's mechanisms for information integration and their far-reaching effects on health, behavior, and society.
